# A retrospective analysis using comorbidity detecting algorithmic software to determine the incidence of International Classification of Diseases (ICD) code omissions and appropriateness of Diagnosis-Related Group (DRG) code modifiers

**DOI:** 10.1186/s12911-024-02724-8

**Published:** 2024-10-23

**Authors:** Eilon Gabel, Jonathan Gal, Tristan Grogan, Ira Hofer

**Affiliations:** 1 University of California at Los Angeles David Geffen School of Medicine, Los Angeles, CA USA; 2 Icahn School of Medicine at Mount Sinai, New York City, NY USA

**Keywords:** International classification of diseases, Diagnosis-related groups, Algorithms, Medical informatics applications, Clinical coding

## Abstract

**Background:**

The mechanism for recording International Classification of Diseases (ICD) and diagnosis related groups (DRG) codes in a patient’s chart is through a certified medical coder who manually reviews the medical record at the completion of an admission. High-acuity ICD codes justify DRG modifiers, indicating the need for escalated hospital resources. In this manuscript, we demonstrate that value of rules-based computer algorithms that audit for omission of administrative codes and quantifying the downstream effects with regard to financial impacts and demographic findings did not indicate significant disparities.

**Methods:**

All study data were acquired via the UCLA Department of Anesthesiology and Perioperative Medicine’s Perioperative Data Warehouse. The DataMart is a structured reporting schema that contains all the relevant clinical data entered into the EPIC (EPIC Systems, Verona, WI) electronic health record. Computer algorithms were created for eighteen disease states that met criteria for DRG modifiers. Each algorithm was run against all hospital admissions with completed billing from 2019. The algorithms scanned for the existence of disease, appropriate ICD coding, and DRG modifier appropriateness. Secondarily, the potential financial impact of ICD omissions was estimated by payor class and an analysis of ICD miscoding was done by ethnicity, sex, age, and financial class.

**Results:**

Data from 34,104 hospital admissions were analyzed from January 1, 2019, to December 31, 2019. 11,520 (32.9%) hospital admissions were algorithm positive for a disease state with no corresponding ICD code. 1,990 (5.8%) admissions were potentially eligible for DRG modification/upgrade with an estimated lost revenue of $22,680,584.50. ICD code omission rates compared against reference groups (private payors, Caucasians, middle-aged patients) demonstrated significant p-values < 0.05; similarly significant p-value where demonstrated when comparing patients of opposite sexes.

**Conclusions:**

We successfully used rules-based algorithms and raw structured EHR data to identify omitted ICD codes from inpatient medical record claims. These missing ICD codes often had downstream effects such as inaccurate DRG modifiers and missed reimbursement. Embedding augmented intelligence into this problematic workflow has the potential for improvements in administrative data, but more importantly, improvements in administrative data accuracy and financial outcomes.

**Supplementary Information:**

The online version contains supplementary material available at 10.1186/s12911-024-02724-8.

**Table:** 

Contributions to Literature
• Computer algorithms are able to automatically audit ICD coding omission by targeting disease states using raw EHR data
• By auditing ICD codes, DRG codes can be evaluated for proper application of severity modifiers automatically
• Insight into the differing rates of ICD code omission among groups by age, sex, payor, and ethnicity

## Introduction

The International Classification of Diseases (ICD) (the most widely used disease catalogue today) originated in 1900 with 180 diseases and has steadily developed into over 120,000 codes [1]. These codes offer incredible specificity allowing healthcare coders to assign precise disease states to patients’ medical records. Beyond good record keeping, ICD codes have become the foundation of disease data for subsequent layers of codes such as the Diagnosis Related Groups (DRG) codes. Developed in 1976, DRG codes are a means to structure the overlapping risk and complexity of patient diagnoses, in-patient medical care and surgical procedures performed in order to arrive at an estimated hospital resource utiliziation [2]. As of 1983, Medicare’s Inpatient Prospective Payment System made ICD and DRG codes the basis for inpatient hospital payments in the United States [3]. 

The recording or ICD and DRG codes from a patient chart is performed by a certified medical coder. At the completion of a hospitalization, coders review the medical record to assign primary and secondary ICD diagnosis codes along with any applicable procedure codes. ICD codes are then used to assign DRG codes via coding algorithms; with high-acuity ICD codes justifying comorbidity modifiers for DRG codes [4]. The Center for Medicare and Medicaid Services (CMS) supports up to three tiers of DRG comorbidity modifiers: base level DRG indicating that patients do not have any complicating diagnoses outside of the reason for hospital admission, Comorbid Condition diagnoses known as “CC,” and Major Comorbid Condition known as “MCC.” [5] The inclusion of CCs or MCCs in the patient’s coded chart shifts the assigned DRG within the group to one that on average justifies increased resource utilization and therefore increased hospital payment to reflect the more resource intensive healthcare needs.

Because of healthcare coding and billing regulation, modern electronic health records (EHR) have adapted tools that allow physicians to document ICD codes within treatment workflows and often require ICD justification when ordering procedures, medications, or laboratory specimens. This is typically done using systematized nomenclature of medicine clinical terms (SNOMED-CT) which allows clinicians to search a disease reference catalogue while storing the associated ICD codes in the EHR [6]. Provider entries are stored in staging areas within the EHR facilitating the process for certified coders to migrate the ICD codes to the subsequent billing schemas [7]. 

Given their structure, ICD codes are used for more than hospital billing. ICD codes are the cornerstone of epidemiologic disease identification in administrative databases and are often used for both Continuous Quality Improvement (CQI) and Health Services Research (HSR). At a national level, payor databases (Medicare or private insurers) and disease specific registries are large repositories with longitudinal patient level data that are frequently indexed by ICD codes. These administrative databases have been heavily used in health economic & outcomes research, precision medicine, quality assessment, hospital & physician report cards, and benchmarking [8–10]. Groups like the Agency for Healthcare Research and Quality (AHRQ) routinely use ICD coding to facilitate the development of clinical practice guidelines [11]. Lastly, these databases are used by payors, such as CMS, to facilitate future reimbursement level for procedures and DRGs [12]. 

Despite the need for accurate ICD coding, many manuscripts have been written about the possible sources of ICD code inaccuracies and have identified sources of error [1, 9, 13–15]. As far back as 1984, the Office of the Inspector General found that 61.7% of inpatient medical records within their study sample had ICD coding errors that favored hospital billing [14]. A subsequent study by the Inspector General found that 14.7% of ICD errors were related to diagnosis codes which would have resulted in payment above the base level DRG [16]. However, both of these studies and many of the other studies that followed have relied on manual clinician chart review to find these coding errors and associated data gaps [17, 18]. While informative about the problems and deficiencies inherent in using administrative data, these studies do little to provide any insight or direction toward a scalable solution.

As an alternative to relying on administrative data, researchers have commonly preferred to use raw clinical EHR data [10]. Using data repositories and intricate disease identifying algorithms, previous manuscripts have been able to demonstrate high fidelity data to assign disease states [19, 20]. Using similar algorithm-based disease identification methods, the gap between clinical and administrative data can be evaluated more closely.

The recent rise of AI, especially neural networks with natural language processing, offers a solution to inaccuracies in ICD coding by automating the documentation of disease states with high accuracy. Neural networks can detect complex patterns in EHRs, reducing human error. However, this comes with a tradeoff: while AI models offer speed and efficiency, they lack the flexibility to fine-tune definitions like rule-based algorithms. As AI systems are often opaque, they may sacrifice coding precision in favor of broader pattern recognition, posing a challenge for ensuring accuracy in clinical settings.

In this manuscript, we hypothesize that rules-based algorithms relying on raw EHR data could be used to audit codes (ICD and DRG) automatically and at scale without the need for manual chart review. As a primary outcome, we evaluate the incidence of missing ICD codes for co-morbid diseases in a cohort of hospital admissions using automated rules-based algorithms based on structured EHR data. Secondarily, we evaluate the extent to which missing ICD codes for the true clinical picture led to an incorrect DRG assignment for the admission, as well as the potential financial impact of these omissions. Lastly, we examine the missing documentation for clinically important demographic associations, especially as it relates to historically underserved groups.

## Materials and methods

### Data extraction

This study (IRB# 15–000518) qualified for IRB exemption status through the UCLA Human Research Protection Program by virtue of having no direct patient contact and using a de-identified dataset. All study data were acquired via a previously published Department of Anesthesiology and Perioperative Medicine at UCLA’s Perioperative Data Warehouse (PDW) [21]. The PDW is a structured reporting data schema that contains all the relevant clinical data entered into the EPIC (EPIC Systems, Verona, WI) electronic health record (EHR). Data are acquired via Clarity, the relational database created by EPIC for data analytics and reporting. While Clarity contains raw clinical data, the PDW was designed to organize, filter, and improve data so that it can be used reliably for creating robust disease phenotypes using clinical logic. Other published manuscripts deriving data from the PDW can be found in the reference Sects. [21–28]. 

Despite the name including the word “perioperative” the DataMart contains data on all UCLA patients regardless of their operative status. It includes data from both the Ronald Reagan UCLA main hospital (500 + beds), Santa Monica UCLA hospital (275 + beds), and all the affiliated outpatient locations.

### Inclusion criteria

Data were extracted for patients who had an inpatient admission to any UCLA health system hospital in calendar year 2019. The rationale for choosing 2019 was to avoid any incomplete billing and only evaluate inpatients that have since been discharged with established DRG designations. Additionally, 2019 was chosen as opposed to 2020 to avoid confounding effects due to the COVID-19 pandemic. If an admission did not have a DRG assigned, or there was no single primary DRG code, then it was excluded from the analysis. Each of these analyses were done at the hospitalization-encounter level so that if a patient was admitted to the hospital multiple times, each admission was treated independently.

### Creating algorithms to identify comorbid diseases

Selected diseases were curated from the CMS list of diseases that met criteria for DRG modifiers. The authors chose diseases that had clear objective criteria that could be mined from EHR data, but it is entirely feasible to create more algorithms that phenotype other disease states. Two of the authors (EG, IH), with experience in designing disease-based algorithms, reviewed the CMS list then came to a consensus with the disease list shown in Table 1 along with the modifier level, ICD codes and structured definitions, and the data sources [19, 22, 29]. The data used for each disease algorithm used mostly clinical data in the form of labs results, echocardiography reads, physician and nursing documentation, medication administrations, operative procedures, and orders. The specific for each individual algorithm are detailed in Table 1. Using this data, each admission was then flagged as having or not having each of the diseases.

**Table 1:** Table of diseases selected for Algorithm Creation with clinical definitions. Table 1 includes a list of the nineteen disease-based algorithms that were coded with clinical definitions. Furthermore, there is a column of ICD codes that represent the clinical disease and equate the use of a DRG modifier. If an admission has any of the listed ICD codes billed, then the billing would be considered appropriate given the clinical condition

Disease	Modifier level	ICD codes	Informatics Rules	Data Source
Acidemia	CC	E71.*, E87.2, E72.*	Any hospital admission with an arterial pH lab result < 7.35.	Lab Results
Bacteremia	CC	R78.81, R65.10,	Any hospital admission with at least two positive blood cultures results during the hospitalization.	Lab Results (cultures)
Blood Loss Anemia	CC	D62	Any hospital admission with a case that has intraoperative blood loss documented as greater than or equal to 300 mL with a corresponding hemoglobin < 12 g/dl within the first 24 h after surgery.	Lab Results Flowsheets
Chronic CHF	CC	I50.*	Any hospital admission with one of: • A documented EF < = 30% by echocardiography within the 12 months prior to admission, • Brain Natriuretic Peptide (BNP) lab value > 900 pg/mL within the 12 months prior to admission • Active medical record problem list of heart failure within the 12 months prior to admission.	Flowsheets Lab Results Past ICD Codes
Chronic Kidney Disease	CC	N18.4, N18.5, N18.8, N18.9	Any hospital admission with a Glomerular Filtration Rate (FGR) < = 30 by using the MDRD calculation.	Lab Results
Delirium	CC	F05, R41.4, R40.3	Any hospital admission with a positive Confusion Assessment Method (CAM) score as documented by ICU nursing.	Flowsheets
Extremes of BMI	CC	E66.2, Z68.1, Z68.4, R64	Any hospital admission with a patient having a recorded BMI > 40 or < 19.	Flowsheets
HIV	CC	B20	Any hospital admission with a historic positive HIV screen or active Zidovudine medication.	Lab Results Medication Orders
Hyponatremia	CC	E87.1	Any hospital admission with a resulted serum amylase sodium value < = 134 mmol/L during the hospitalization.	Lab Results
Myocardial injury	CC	S26.90XA, I25.*, I24.8, I24.9	Any hospital admission with a positive troponin lab result.	Lab Results
Transplant	CC	T86.*, Z94.*	Any hospital admission with a historical transplant surgery or tacrolimus medication administration.	Surgery Logs Medication Orders
UTI	CC	N39.0, O23.40, O23.41, O86.2, O03.38, O04.88, O08.83, O23.41, O23.42, O23.43	Any hospital admission with a bacterial urine culture result showing at least 100,000 colonies.	Lab Results (cultures)
Acute MI	MCC	I21.*, I22.*	Any hospital admission that has a positive troponin lab result during the admission and has a coronary stent implanted during the admission (from CPT codes).	Lab Results Surgery Logs
Acute Pancreatitis	MCC	K85.*	Any hospital admission with either: • a serum amylase lab result > 1000 U/L • serum lipase lab result > 450 U/L.	Lab Results
Altered Mental Status	MCC	R40.*	Any hospital admission with a Glasgow Coma Scale (GCS) < = 8 entered by nursing staff.	Flowsheets
End Stage Renal Disease	MCC	N18.6, N17.0, N17.1,	Any hospital admission with documentation of dialysis flow and volumes using flowsheet data or ultrafiltration data as entered by dialysis technicians.	Flowsheets
Inpatient death	MCC		Any hospital admission resulting in an inpatient death.	Admission Logs
Respiratory Failure	MCC	J96.*, J95.*	Any hospital admission with a documented endotracheal tube or high flow nasal cannula (HFNC) and no surgical procedures done during the index admission.	Surgery Logs Flowsheets Airway Documentation
Severe Malnutrition	MCC	E40.*, E41.*, E42.*, E43.*	Any hospital admission with documentation of total parenteral nutrition (TPN).	Medication Orders

### Identifying cases with missing administrative data

For each of the eighteen disease algorithms, a global list of ICD was generated by looking at historic hospital admission that satisfied the algorithm logic. Then the list was manually reviewed to only include the ICD codes that were clinically relevant to the specific algorithm and qualify for DRG modifiers. In an example, the algorithm evaluating myocardial injury was satisfied by ICD codes for myocardial infarction, acute ischemic disease, or unspecified cardiac injury. The final list of ICD codes is found in Table 1 with accompanying algorithm definitions. Admissions that had clinical evidence of the disease in the algorithm but did not have any of the selected ICD codes were flagged as missing applicable ICD data.

### Categorizing the diagnosis related groups (DRGs)

A complete list of DRG codes was downloaded from the Center for Medicare and Medicaid Services (CMS) website: www.CMS.gov. Within the downloaded file, data for each DRG included the DRG value and a text description.

After importing the DRG data into the PDW database, each unique DRG was designated a comorbidity level; “MCC”, “CC”, “Base”, or “Singlet.” While some DRG codes are “Doublets” indicating that there are only 2 possible states, they typically have a “Base/CC” and “CC/MCC” designation and modification is still partly allowable. MCC indicated that the DRG included a major comorbid condition (MCC) which was triggered from the text description in the CMS data containing the phrase “WITH MCC” at the end of the description such as “PLEURAL EFFUSION WITH MCC.” The same applied for DRG’s with the comorbid condition (CC) designation. These code descriptions ended with the phrase “WITH CC’’ such as “PLEURAL EFFUSION WITH CC.” For codes where the description included verbiage that excluded MCC or CC levels, such as “PLEURAL EFFUSION WITHOUT CC/MCC’’, we assigned the “Base” level. This implied that the same diagnosis DRG had the ability to scale upwards. Lastly, the “Singlet” designation was given to codes that had no indication of the presence of CC/MCC modifiers and had no codes with the same descriptors to indicate the ability to increase or decrease the level of complexity.

Once the codes were classified using discrete column variables to indicate comorbidity level, all the text modifiers were stripped out of the text descriptions. For example, “PLEURAL EFFUSION WITH MCC”, “PLEURAL EFFUSION WITH CC,” and “PLEURAL EFFUSION WITHOUT CC/MCC” where all grouped to “PLEURAL EFFUSION’’ and classified as a single disease state. This approach allowed us to identify diagnoses where there exists the ability to change the DRG based on the presence or absence of relevant comorbidities.

### Evaluating DRGs for modifiers

When an algorithm detected the presence of a disease state, and the billed DRG comorbidity level was below the comorbidity level of the algorithm disease comorbidity, a flag was assigned to the admission to indicate an inaccurate comorbidity level was coded since the missing ICD code would have justified a higher DRG modifier. For example, admissions that were flagged by the acute pancreatitis algorithm (MCC worthy), but only had existing DRG codes with base or CC level modifiers, were flagged as appropriate for MCC designation since pancreatitis was appropriate based on conservative limits of clinical laboratory data. There are three possible upgrade types: base to CC, CC to MCC, and base to MCC. In the event that a different disease algorithm flagged an admission for comorbidity level modification, the highest DRG modifier level was used; essentially eliminating redundancy. If an admission DRG was given the “Singlet” designation, then there was no consideration for possible modification. This process was applied to each hospitalization encounter.

### Categorizing hospital payors

Payors were classified into three categories: Medicare, Medi-Cal, and Private. The payor grouping was done manually by evaluating the 2019 payors in the EMR by financial class. Medi-Cal and Medicaid were consolidated to Medi-Cal, while Medicare and Medicare Advantage were consolidated to Medicare. All the other payor types were grouped into the private category.

### Incorporating weighting factors

Using the CMS website, we downloaded data on the 2019 DRG Relative Weighting Factors (RWF) [24]. RWF are used in hospital billing to assign hospital reimbursement to DRG values, essentially putting a monetary value to each DRG based on resource intensity. In practice different payors negotiate and contract assigning dollar amounts to RWF (in fact this may also differ by provider and insurance line of business for a given payor). For analysis purposes, we estimated that a single RWF point was worth $20,000 for private payors, $10,000 for Medicare, and $7,500 for Medicaid (Medi-Cal). These values were informed by our previous experience using RWF [30].

When evaluating hospital admissions that were flagged for upgrade by the disease algorithms, we calculated the delta RWF between the originally billed DRG, and the algorithm proposed upgraded DRG with modifiers. When multiplying the delta RWF by the estimated payor-based RWF value, we were able to derive the lost revenue for not coding the higher level DRG.

### Statistical methods

Incidence rates were computed for each condition for both primary and secondary outcomes (ICD/DRG) and 95% confidence intervals were constructed using the binomial distribution. Analyses were conducted using R v4.1.0.

In looking for findings did not indicate significant disparities between age, sex, and payor, these data were analyzed using two methods, Standardized Mean Difference (SMD) and direct comparison of each group to a reference group within each category.

*SMD*: Given the large sample size, the analysis is overpowered in determining significant differences. In other words, a 1% difference would be statistically significant with little clinical value. Published guidelines for interpreting the magnitude of the SMD in the social sciences include: small, SMD 0-0.4; medium, SMD 0.4–0.6; and large, SMD 0.6-1.0 [25].

*Reference Groups*: Reference groups were chosen from the race, payor, and age categories to compare the incidence of ICD miscoding within each category. The index groups were Caucasians (non-Hispanic), private payors, and middle-aged patients. Each comparison was then represented by a p-value with values < 0.05 being statistically significant.

### De-identification

While the algorithm scripts were written using the PDW which houses identified data, all research extraction was done using de-identification methods that meet the standards of the UCLA health system. Any unnecessary identifier was removed from the data and any necessary linkages were subjected to a hash function using an random salt that is unique to each patient. Lastly, all dates were modified to be relative values starting at the first encounter date that each patient had in the EMR data.

## Results

Data were analyzed from January 1, 2019, to December 31, 2019. In total, 34,982 hospitalizations met inclusion criteria for having DRG assignment data with 34,104 (97.5%) of the admissions having complete data and a primary DRG designated. 5,388 (15.4%) of the DRG codes were at the base comorbidity level, 13,227 (37.8%) had a CC modifier, 12,591 (36%) had a MCC modifier, and 3,776 (10.8%) had no applicable modifier and were considered “Singlet.” These data are summarized in a supplemental table.

### Application of algorithms

The results of how many admissions (individual hospitalization encounters) were flagged by each disease algorithm and the count of applicable ICD codes can be found in Table 2. In total, 11,520 (32.9%) hospital admissions were flagged by our algorithms as having a disease state with no corresponding ICD code in the EHR. Among these flagged cases, some of the most notable were Acidemia with 63.2% of cases missing the appropriate ICD code and Delirium, which had an even higher omission rate of 80.3%. Hyponatremia also had a significant percentage of omissions, with 62.3% of cases missing ICD codes.

**Table 2:** Incidence of Admission Identification by Algorithm with incidence of Unbilled ICD codes and Inappropriate DRG modifiers. Table 2 demonstrates the incidence of admissions that meets criteria for each of the nineteen disease algorithms as described in table 1. The table also demonstrates the incidence of algorithm-identified admissions that are missing acceptable ICD codes. Furthermore, of the algorithm-identified admissions, the incidence of inadequate DRG modifiers, relative to the algorithm disease state are shown. Lastly, the table indicates the number of occurrences where the ICD codes were available, but the disease states did not meet the algorithms’ criteria for inclusion. Note that patients in each row can be represented multiple times making the total larger than the counts of patients meeting criteria

	Count of Admissions Meeting Disease Algorithm / Clinical Criteria	Count of Admissions Meeting Algorithm / Clinical Criteria *without Disease-Specific ICD Codes*	Count of Admissions Meeting Algorithm / Clinical Criteria *with Inappropriate DRG Modifiers*	Count of Admission with Disease-Specific ICD Codes Where Algorithm Was Not Sensitive to Detect Disease
Acidemia	2,453	1,551 (63.2%)	73 (3%)	2,055 (45.6%)
Acute MI	32	4 (12.5%)	8 (25%)	2,010 (98.4%)
Bacteremia	774	195 (25.2%)	11 (1.4%)	1,093 (59.5%)
Chronic CHF	1,363	293 (21.5%)	33 (2.4%)	4,374 (76.2%)
CKD	4,325	1,061 (24.5%)	90 (2.1%)	3,915 (47.5%)
Death	946		23 (2.4%)	
Delirium	710	570 (80.3%)	14 (2%)	644 (47.6%)
Extreme BMI	3,931	1,840 (46.8%)	398 (10.1%)	1,087 (21.7%)
GCS < = 8	3,457	1,715 (49.6%)	633 (18.3%)	3,420 (49.7%)
Hemodialysis	539	86 (16%)	15 (2.8%)	2,805 (83.9%)
HIV	299	21 (7%)	10 (3.3%)	65 (17.9%)
Hyponatremia	9,772	6,091 (62.3%)	431 (4.4%)	196 (2%)
Myocardial Injury	2,631	977 (37.1%)	92 (3.5%)	5,321 (66.9%)
Pancreatitis	225	45 (20%)	69 (30.7%)	277 (55.2%)
Post-op anemia	1,138	304 (26.7%)	61 (5.4%)	4,016 (78%)
Respiratory Arrest	854	33 (3.9%)	29 (3.4%)	4,580 (84.3%)
Severe Malnutrition	794	460 (57.9%)	157 (19.8%)	1,775 (70%)
Transplant	2,801	112 (4%)	50 (1.8%)	594 (17.5%)
UTI	2,065	600 (29.1%)	52 (2.5%)	1,716 (45.4%)
Total	39,120	13,313 (34%)	2,368 (6.1%)	39,943 (50.5%)

In cases where the originally assigned DRG comorbidity modifier level was below the algorithm-derived comorbidity level, the hospital admission was flagged as not having adequate ICD codes specifically assigned to the index hospitalization. The results of the DRG upgrades per disease state are also demonstrated in Table 2. For instance, Pancreatitis had the highest percentage of cases flagged for potential DRG upgrade, with 30.7% of cases identified as needing a change in the comorbidity modifier. The GCS ≤ 8 category was also notable, with 18.3% of admissions requiring a DRG upgrade.

**Fig. 1:**
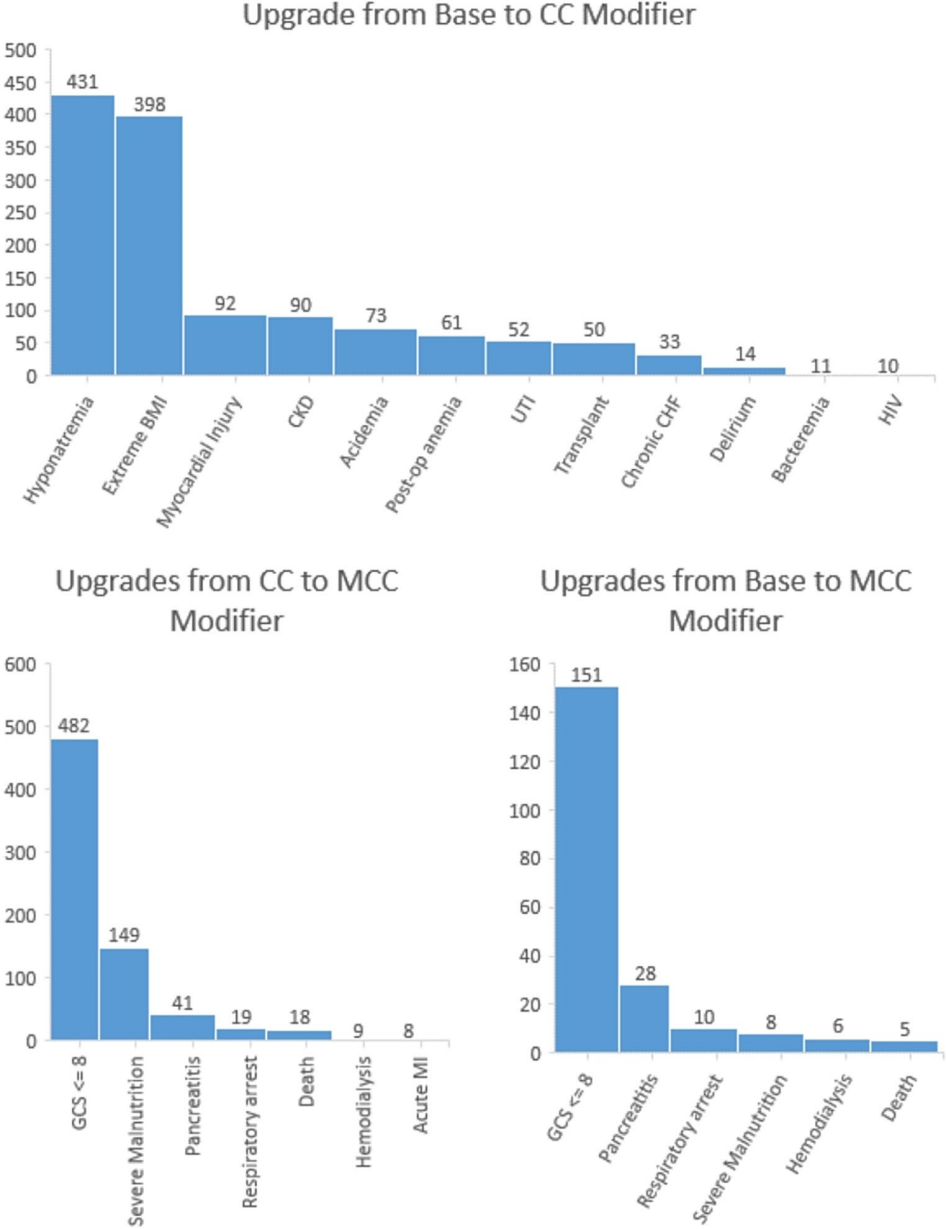
Distribution of DRG Upgrade Eligibility by Algorithm Type Fig. 1 illustrates the incidence of admissions with DRG modifiers that are at a lower acuity than the disease states associated with the applicable algorithms. Only cases that can justify upgrading to Comorbid Condition (CC) or Major Comorbid Condition (MCC) are shown

The DRG upgrades per disease state, also demonstrated in Table 2, showed that 1,127 (3.2%) admissions were flagged for upgrade from the base level to CC level, 185 (0.53%) admissions were flagged for upgrade from the base level to MCC level, and 678 (1.9%) admissions were flagged for upgrade from CC to MCC level. The breakdown of how many upgrades were from base to MCC level vs. CC to MCC level is demonstrated in Fig. 1. Hyponatremia was flagged for the highest number of potential upgrades, with 431 cases (4.4%) needing a higher DRG modifier. Lastly, Table 2 demonstrates the specificity of the algorithms by indicating the number of occurrences where the ICD codes were available, but the disease states did not meet the algorithms’ criteria for inclusion. For example, Acidemia had 2,055 cases (45.6%) where ICD codes were present but did not meet the algorithm’s criteria, showing the importance of this approach in highlighting potential misclassifications.

### Accounting for potential revenue optimization

Accounting for the difference in RWF points between the DRG codes originally assigned to admission and the DRG codes assigned by the algorithm, we were able to create the delta RWF values. Multiplying the delta RWF values by the payor-dependent dollar estimated per point, we calculated the total lost revenue by admission due to miscoding. In total, we found that the value of miscoding DRGs amounted to a loss of $22,680,584.50. $4,971,278 was attributed to upgrades from the base to CC comorbidity levels, $2,480,785 was attributed to upgrades from the base to MCC comorbidity levels, and $15,228,522 was attributed to upgrades from the CC to MCC comorbidity levels. A financial summary is available in Table 3 where the data is also broken down by payor.

**Table 3:** Potentially lost relative weighting factors from inadequate DRG modifiers. Summation of DRG relative weighting factors (RWF) between billed DRG codes and algorithm-proposed DRG codes with modifiers. Estimation of revenue loss was calculated based on payor-specific estimated dollars per RWF

Upgrade Type	Payor	Count	Added points	$/Unit	Additional Revenue
Upgrade Base to CC	MEDI-CAL	254	75.4941	$7,500.00	$566,205.75
	MEDICARE	198	72.7078	$10,000.00	$727,078.00
	PRIVATE	583	183.8997	$20,000.00	$3,677,994.00
Upgrade Base to MCC	MEDI-CAL	87	133.8745	$7,500.00	$1,004,058.75
	MEDICARE	75	54.3528	$10,000.00	$543,528.00
	PRIVATE	32	46.6599	$20,000.00	$933,198.00
Upgrade CC to MCC	MEDI-CAL	134	201.6356	$7,500.00	$1,512,267.00
	MEDICARE	296	376.6575	$10,000.00	$3,766,575.00
	PRIVATE	355	497.484	$20,000.00	$9,949,680.00
					$22,680,584.50

### Demographic association

Demographic information for the hospitalizations flagged by the algorithms for not having a proper ICD code applied to the chart is summarized in Table 4. When using the standardized mean difference (SMD) calculated for each table row, as described in the statistical method section, they all fall into the small association category (SMD < = 0.4). This indicates that there is minimal association and little clinical relevance. The data in the table did not restrict the number of ICD codes for payor maximums and used all the codes entered in the EMR for the admission.

**Table 4:** Evaluation of the standardized Mean difference for the outcome of proper ICD billing broken down by sex, ethnicity, Financial Group, and Age. Using the standardized Mean difference (SMD), table 5 demonstrates differences in sex, ethnicity, payor, and age in the cohorts for admissions with accurate ICD coding and those with unbilled ICD codes. All the SMD values were in the low difference group indicating that there are unlikely any disparities. Additionally, setting reference categories (caucasian, middle-aged, private patients) direct comparisons were done with resulting P values. This data includes all ICD codes in the EMR without restricting to payor maximums

	Accurate ICD Coding (24,621)	Unbilled ICD Coding (10,361) 29.6%	SMD	*p*-value
**Sex**			0.168	< 0.001
Male	10,739 (43.6%)	5385 (52.0%)		
Female	13,882 (56.4%)	4976 (48.0%)		
**Race / Ethnicity**			0.085	< 0.001
Asian/Pacific Islander	2406 (9.8%)	1004 (9.7%)		0.112
Black	2319 (9.5%)	1099 (10.7%)		< 0.001
Caucasian-Hispanic	1824 (7.4%)	847 (8.2%)		< 0.001
Caucasian-Non-Hispanic	12,824 (52.3%)	5041 (48.9%)		REF
Other-Hispanic	3095 (12.6%)	1496 (14.5%)		< 0.001
Other-Non-Hispanic	2057 (8.4%)	831 (8.1%)		0.52
**Payor Group**			0.243	< 0.001
Medi-Cal	3329 (13.5%)	1570 (15.2%)		< 0.001
Medicare	8689 (35.3%)	4709 (45.4%)		< 0.001
Private	12,603 (51.2%)	4082 (39.4%)		REF
**Age Group**			0.219	< 0.001
18–35 (young adult)	5703 (24.2%)	1577 (15.9%)		< 0.001
36–64 (middle age)	9945 (42.1%)	4300 (43.5%)		REF
65–78 (aged)	5432 (23.0%)	2816 (28.5%)		< 0.001
79+ (old)	2534 (10.7%)	1199 (12.1%)		0.033

Alternatively, the p-value generated by comparison of each patient group to the reference groups (Caucasians non-Hispanic, private payors, and middle aged) all demonstrated significant p-value < 0.001 with the exception of Asian patients, patients > 79 years old, and patients with ethnicity listed as “other – non Hispanic.”

### ICD limits

In some cases, payors will limit the number of ICD codes that the hospital is able to submit. This requires billers to choose the most optimal ICD codes to maximize revenue while also ensuring accurate account of the hospitalization. In our data, 6,493 Medicare cases met the maximal 25 ICD codes limit. Of those, 156 where eligible for un upgrade for one of the three upgrade types. It’s unclear which of the ICD were ultimately submitted as the filtration can occurs downstream of our system.

## Discussion

In this manuscript, we successfully used rules-based algorithms and raw structured EHR data to identify ICD codes that were not coded in patients’ medical record claims for completed hospitalizations. These missing ICD codes often had downstream effects such as incorrect DRG modifiers and ultimately incorrect reimbursement. Overall, 34% of hospital admissions were deemed as missing an ICD code and 5.8% of admissions had miscoded DRG modifiers. 0.6% of the miscoded DRG codes were eligible for MCC modifiers despite not even having a CC modifier. This resulted in an estimated $22,680,584.50 USD of unbilled revenue over the course of a single year for this study. These results indicate there may be value in using these kinds of algorithms to identify the 19 disease states that were the focus to improve medical coding and enhance ICD/DRG accuracy.

In this study, our goal was not to fully assess the accuracy or inaccuracy of all ICD codes, but rather to test the hypothesis that rules-based algorithms can be used to identify hospital admissions that *might* be eligible for upward ICD adjustments and improved billing and / or payment. For an ICD code to be eligible for billing, the disease must not only be present but also addressed by a treating provider. The algorithms here only identify that the *clinical* criteria were present, not that the necessary chart documentation of management and / or treatment was also present. For example, a patient that had hyponatremia by laboratory results, but no documentation by the provider, was identified by our algorithms as having hyponatremia but is not eligible for increased billing due to a lack of documentation/treatment. This emphasizes that there is still a need for human review followed by clarification of documentation queries to treating providers with the hope to use augmented intelligence to aid in the process. Thus, this manuscript sheds light on discrepancies between ICD codes and the true clinical picture, but the study was not designed to inform the accuracy of the ICD coding as it relates to the necessary clinical documentation.

Considering the differences between clinical presence of a disease and the regulatory requirements for coding, we believe that this study is most reflective of the fact that modern EHRs increasingly seem to have large volumes of relevant data but are often hard to navigate by practicing providers (and administrative staff such as billers & coders). A single hospital admission may generate a multitude of laboratory and radiographic results, and dozens of provider notes. It is not possible for a human to review this magnitude of data on their own. We believe that automated computer-based algorithms, such as those used here, can be a potential part of the solution.

At the outset of our study, we hypothesized that the missing diagnoses might be based on patient demographics. Issues of systemic bias in healthcare are now well documented [31–33]. It is certainly plausible that these systemic issues resulted in providers being more likely to miss key conditions in some patients as opposed to others. Looking at the standard mean difference (SMD), all the variation was in the minimal difference range. However, when comparing the frequencies of groups with missing ICD coding against reference groups (private payors, Caucasians, middle-aged patients) we were able to derive statistically significant p-values. Similarly significant p-value where demonstrated between patients of opposite sexes. The only comparisons that were not significant included Caucasian vs. Asian patients, Caucasian vs. “other non-Hispanic” patients, and middle-aged vs. elder patients. Given the discrepancy between the significant p-values using reference groups and a small deference by SMD, this raises the concern of having statistical significance without clinical significance and far more research is necessary to understand these implications.

Inaccurate or incomplete ICD coding have a significant impact on a health system beyond direct financial reimbursement. Given their widespread use, and assumptions of accuracy, hospital leadership, regional groups, payers and even the federal government routinely use ICD codes for risk adjustment purposes and researchers often use ICD codes in retrospective studies examining healthcare utilization, patient outcomes, and similar metrics [34–36]. As healthcare transitions towards value-based care paradigms these secondary uses are likely to take on increased importance.

There are limitations to this study. First, this is a single center retrospective analysis carried out at a large academic medical center. The results here may not be directly applicable to all other hospitals and certainly might be different in centers with different levels of acuity. It is also important to note that many assumptions were made to provide a clear analysis – these assumptions may not always be perfect for a given patient. Thus, the exact numbers cited in this manuscript may not be 100% accurate; however, we believe the overall conclusions are correct. There are also exceptions where certain ICD codes do not qualify for modification of a DRG like in the example of morbid obesity as it pertains to a DRG for bariatric surgery since the obesity is implied – these exclusions were not accounted for in our analysis and may affect the final rates that were reported. Nonetheless, given the magnitude of missing ICD codes and potential for changes in DRGs we believe that the results certainly demonstrate room for improvement. Secondly, the financial analysis that was used to estimate the lost annual revenue from the selected disease algorithms was only an estimate. Reimbursement for the relative weight factors (RWF) were estimated based on insurance categorization. To derive a more accurate number, work would need to be done in conjunction with billing data to arrive at a more specific insurer/policy reimbursement rate. Also, over the course of the study, factors such as inflation and other reimbursement changes occur naturally over time which were not accounted for in our financial modeling. Lastly, despite the algorithms indicating existence of a disease, it’s possible that the disease did not receive any treatment during the given hospitalization and thus does not warrant billing consideration. This suggests that algorithms have the potential to “up code” for disease states that do not justify ICD coding. We believe that the algorithms are specific enough to indicate active disease states that are being addressed.

In trying to create a more accurate picture of ICD / DRG coding and actual patient disease burden, these algorithms fail in identifying patients that are assigned codes that are at a higher acutely then appropriate. While technically possible, the algorithms were designed to identify diseases in a “rule-in” approach rather than “rule-out.” This is something we plan to address in future works.

Despite these limitations, we believe this manuscript demonstrates that algorithms based on raw EHR data can be used to identify disease states. Similarly, this manuscript adds to the existing body of work demonstrating issues with relying on ICD codes as a complete picture of a given patient’s clinical risk. The implementation of systems, like these algorithms, can likely have benefit for clinician staff in the form of clinical decision support and for the coding teams as admission summaries. Embedding augmented intelligence into this problematic workflow has the potential for improvements in administrative data, but more importantly, improvements in administrative data accuracy and financial outcomes.

## Conclusion

We successfully used rules-based algorithms and raw structured EHR data to identify omitted ICD codes from inpatient medical record claims. Using a short list of eighteen diseases, 34% of the algorithm positive hospital admissions were identified as having an ICD code omission and 5.8% of the admissions had miscoded DRG modifiers. Using these automatable algorithms can help augment the coding process to improve the accuracy of ICD coding and potentially minimize findings did not indicate significant disparities.

## Electronic supplementary material

Below is the link to the electronic supplementary material.

Supplementary Material 1

## Data Availability

Given that the data used for this study is property of UCLA, it is not available for sharing publicly. Furthermore, the code is written based on a proprietary system and cannot be shared readily in the current form without possible copyright issues from the EHR vendor.

## Abbreviations

ICD: International Classification of Diseases
DRG: Diagnosis Related Groups
CMS: The Center for Medicare and Medicaid Services
HER: Electronic Health Records
PDW: UCLA’s Perioperative Data Warehouse
MCC: Major Comorbid Condition
CC: Comorbid Condition
RWF: Relative Weighting Factors
SMD: Standardized Mean Difference
