# Supplementary material for: A retrospective analysis using comorbidity detecting algorithmic software to determine the incidence of International Classification of Diseases (ICD) code omissions and appropriateness of Diagnosis-Related Group (DRG) code modifiers
Source: BMC Med Inform Decis Mak. 2024 Oct 23;24:309. doi: 10.1186/s12911-024-02724-8 (PMC11520144; doi:10.1186/s12911-024-02724-8)
Supplement: Supplementary file 1 — Supplementary Material 1 [file 12911_2024_2724_MOESM1_ESM.docx]

**Supplemental Table:
 Title: Table of Admission Demographic Information Divided by DRG Modifiers**

**Caption: Table 2 breaks down the patient cohorts by the different DRG modifier levels (i.e. Base, CC, MCC) with supplemental demographics such as age and sex. Furthermore, there is a subdivision of ethnicity and payor grouping.**

|  |  | BASE DRG | | CC DRG | | MCC DRG | | Singlet DRG | |
| --- | --- | --- | --- | --- | --- | --- | --- | --- | --- |
| Demographics | Total Admissions | 5388 | 15.4% | 13229 | 37.8% | 12588 | 36% | 3777 | 10.7% |
|  | Average Age | 45.5 |  | 55.2 |  | 59.5 |  | 49.53 |  |
|  | Male sex | 1607 | 30% | 6080 | 46% | 6619 | 52% | 1818 | 48% |
|  | Female Sex | 3781 | 70% | 7149 | 54% | 5969 | 48% | 1959 | 52% |
| Ethnicity | UNKNOWN | 28 | 0.5% | 53 | 0.4% | 47 | 0.4% | 16 | 0.4% |
|  | BLACK | 380 | 7.1% | 1239 | 9.4% | 1418 | 11.3% | 391 | 10.4% |
|  | CAUCASIAN-HISPANIC | 442 | 8.2% | 1010 | 7.6% | 889 | 7.1% | 299 | 7.9% |
|  | OTHER-NOT HISPANIC | 512 | 9.5% | 1047 | 7.9% | 1006 | 8% | 305 | 8.1% |
|  | OTHER-HISPANIC | 699 | 13% | 1753 | 13.3% | 1726 | 13.7% | 448 | 11.2% |
|  | ASIAN/PACIFIC ISLANDER | 677 | 12.6% | 1190 | 9% | 1194 | 9.5% | 356 | 9.4% |
|  | CAUCASIAN-NOT HISPANIC | 2650 | 49.2% | 6937 | 52.4% | 6308 | 50.1% | 1962 | 51.9% |
| Payor Group | MEDI-CAL | 741 | 13.8% | 1919 | 14.5% | 1834 | 14.6% | 405 | 10.7% |
|  | MEDICARE | 812 | 15.1% | 5021 | 38% | 6189 | 49.2% | 1376 | 36.4% |
|  | PRIVATE | 3835 | 71.2% | 6289 | 47.6% | 4565 | 36.2% | 1996 | 52.8% |
